# A statistical approach for tracking clonal dynamics in cancer using longitudinal next-generation sequencing data

**DOI:** 10.1093/bioinformatics/btaa672

**Published:** 2020-07-28

**Authors:** Dimitrios V Vavoulis, Anthony Cutts, Jenny C Taylor, Anna Schuh

**Affiliations:** 1 Department of Oncology, University of Oxford, Oxford, OX3 7DQ, UK; 2 Nuffield Department of Medicine, Wellcome Centre for Human Genetics, University of Oxford, Oxford, OX3 7BN, UK; 3 NIHR Oxford Biomedical Research Centre, Oxford University Hospitals NHS Trust, Oxford, OX3 9DU, UK; 4 Department of Oncology, Molecular Diagnostic Centre, University of Oxford, Oxford OX3 9DU, UK; 5 Department of Haematology, Oxford University Hospitals NHS Trust, Oxford OX3 9DU, UK

## Abstract

**Motivation:**

Tumours are composed of distinct cancer cell populations (*clones*), which continuously adapt to their local micro-environment. Standard methods for clonal deconvolution seek to identify groups of mutations and estimate the prevalence of each group in the tumour, while considering its purity and copy number profile. These methods have been applied on cross-sectional data and on longitudinal data after discarding information on the timing of sample collection. Two key questions are how can we incorporate such information in our analyses and is there any benefit in doing so?

**Results:**

We developed a clonal deconvolution method, which incorporates explicitly the temporal spacing of longitudinally sampled tumours. By merging a Dirichlet Process Mixture Model with Gaussian Process priors and using as input a sequence of several sparsely collected samples, our method can reconstruct the temporal profile of the abundance of any mutation cluster supported by the data as a continuous function of time. We benchmarked our method on whole genome, whole exome and targeted sequencing data from patients with chronic lymphocytic leukaemia, on liquid biopsy data from a patient with melanoma and on synthetic data and we found that incorporating information on the timing of tissue collection improves model performance, as long as data of sufficient volume and complexity are available for estimating free model parameters. Thus, our approach is particularly useful when collecting a relatively long sequence of tumour samples is feasible, as in liquid cancers (e.g. leukaemia) and liquid biopsies.

**Availability and implementation:**

The statistical methodology presented in this paper is freely available at github.com/dvav/clonosGP.

**Supplementary information:**

Supplementary data are available at *Bioinformatics* online.

## 1 Introduction

Cancer cells undergo a process of Darwinian evolution in response to selective pressures in their local micro-environment, for example, as a result of therapeutic intervention (Merlo *et al.*, 2006; Nowell, 1976). This induces cell propagation and diversification during tumour growth, which result in a heterogeneous population of phylogenetically related, but genotypically and phenotypically distinct cancer cell populations, known as *clones*. Tumour heterogeneity is clinically important because it complicates the molecular profiling of tumours and enables the fittest cancer cells to escape treatment leading to relapse. Monitoring this process of continuous adaptation requires a detailed characterization (through the use of next-generation sequencing, bioinformatics and statistical analysis) of the somatic aberrations harboured by the tumour at various time points over the course of the disease.

A major challenge in solving the problem of *clonal deconvolution* using bulk sequencing data is the fact that tumour heterogeneity is not directly observed, but rather inferred through the analysis of samples, each of which is a mixture of normal and cancer cells from various clones. Despite (or because of) this, clonal deconvolution has been the subject of much statistical innovation (see Beerenwinkel *et al.*, 2015; Dentro *et al.*, 2017; Ismail *et al.*, 2019; Salcedo *et al.*, 2020 for a review). Current statistical methodologies seek to identify the number of clones in a tumour, their somatic mutation content, prevalence and phylogenetic relations and they can be used for the analysis of cross-sectional data (obtained, for example, through multiple biopsies from the same patient) or longitudinal data after discarding any information on the timing of tissue sample collection (Abécassis *et al.*, 2019; Deshwar *et al.*, 2015; Donmez *et al.*, 2017; El-Kebir *et al.*, 2015; Fischer *et al.*, 2014; Jiang *et al.*, 2016; Marass *et al.*, 2016; Miller *et al.*, 2014; Myers *et al.*, 2019; Roth *et al.*, 2014; Rubanova *et al.*, 2018; Sengupta *et al.*, 2014; Yuan *et al.*, 2015, 2018; Zare *et al.*, 2014; Zucker *et al.*, 2019).

In this article, we pose the following two questions: (i) how can we incorporate temporal spacing information in the analysis of sequentially collected samples (typically over several months or years) and (ii) is there any benefit in doing so? We begin with a standard Bayesian non-parametric model for clustering somatic mutations with similar observed frequencies, while simultaneously correcting for sample purity and local copy number variation. We extend this model by treating the cluster prevalences as functions of time, which follow a Gaussian process prior. The advantage of this approach is that we do not need to impose a particular functional form on the time dependence of cluster abundances, but only some general properties (e.g. smoothness, amplitude and time scale), which are estimated from the data. In return, we obtain a continuous reconstruction of the time course of each cluster during the course of the disease from a small number of sequentially collected samples. We test various model configurations on whole genome sequencing (WGS), whole exome sequencing (WES) and targeted genome sequencing (TGS) data from patients with chronic lymphocytic leukaemia (CLL; González-Rincón *et al.*, 2019; Schuh *et al.*, 2012), on data from the liquid biopsy of a patient with melanoma (Cutts *et al.*, 2017) and on synthetic data, and we demonstrate that incorporating temporal information in our analysis can boost the performance of clonal deconvolution.

## 2 Materials and methods

We present a series of models of increasing complexity starting with the statistical model for a single tumour sample.

### 2.1 Model for a single tumour sample

We assume that a tumour has been sequenced at *N* bi-allelic genomic loci harbouring somatic mutations. For each locus *i*, we can calculate the observed *variant allele fraction* (VAF) as the ratio $r_{i}/(r_{i}+r_{i}^{\mathrm{ref}})$, where *r_i_* and $r_{i}^{\mathrm{ref}}$ are the number of reads harbouring the alternative and reference alleles, respectively. The expected value *θ_i_* of the VAF for mutation *i* is a function *f* of the *cancer cell fraction* (CCF), i.e. the fraction ${\tilde{\phi }}_{i}$ of cancer cells that harbour the mutation, $\theta_{i}=f({\tilde{\phi }}_{i})$. The population of cancer cells is partitioned in a finite, but unknown, number of clones, each harbouring a unique set of mutations. This implies that different mutations share the same CCF value, i.e. the mutation-specific fractions ${\left\{{\tilde{\phi }}_{i}\right\}}_{i=1}^{N}$ are not all distinct. We model this structure with a *Dirichlet Process* prior on ${\tilde{\phi }}_{i}$ with concentration parameter *α* and a uniform base distribution $G_{0}\equiv U(0,1)$ (Gelman *et al.*, 2013; Nik-Zainal *et al.*, 2012). Using the stick-breaking representation of the Dirichlet Process, ${\tilde{\phi }}_{i}$ is modelled as an infinite mixture, as shown below:

$$\begin{array}{c} {\tilde{\phi }}_{i}∼\sum_{k=1}^{\infty }w_{k}\delta_{\phi_{k}}({\tilde{\phi }}_{i})\;\;\phi_{k}∼G_{0}\;\;u_{k}∼B(1,\alpha )\\ w_{1}=u_{1}\;\;w_{k}=u_{k}\prod_{l=1}^{k-1}\left(1-u_{l}\right)\;\;\frac{1}{1+\alpha }∼U(0,1) \end{array}$$

where $\delta_{\phi_{k}}(\cdot )$ is the Dirac delta function centred at $\phi_{k}$ and B(·,·) indicates a beta distribution. The uniform prior on the mean of the beta function ${\left(1+a\right)}^{-1}$ implies that the prior on the concentration parameter is $\alpha ∼{\left(1+\alpha \right)}^{-2}$, which is similar to the standard exponential distribution, but with higher kurtosis, resulting in a heavier tail. In practice, we approximate the above infinite sum by truncating at a large value *K* (Ishwaran and James, 2001). Here, we take *K *=* *20, which is more than twice the largest number of estimated clusters across all examined datasets (see Section 3; Fig. 4).

### 2.2 Joint model for clonally related tumour samples

The above model can be extended to multiple clonally related samples by allowing the CCF variables to vary between samples (Bolli *et al.*, 2014; Roth *et al.*, 2014). For *M* samples (and truncation *K*), we have:

$${\left\{{\tilde{\phi }}_{ij}\right\}}_{j=1}^{M}∼\sum_{k=1}^{K}w_{k}\prod_{j=1}^{M}\delta_{\phi_{jk}}({\tilde{\phi }}_{ij})\;\;\phi_{jk}∼U(0,1)$$

where the rest of the model remains the same as for the one-sample case. Effectively, we incorporate multiple samples in the model by allowing the cluster centres $\phi_{jk}$ to vary across samples. As a prelude to the next section, we note that the transformed variable $\psi_{jk}=\text{log}\;\phi_{jk}-\text{log}\;(1-\phi_{jk})$ follows a standard logistic distribution, $\psi_{jk}∼\text{Logistic}(0,1)$. Below, instead of the logistic distribution, we use a parameterized multivariate normal distribution, as explained in more detail in the next section.

### 2.3 Single-output Gaussian process model for longitudinal tumour samples

The above model does not consider the temporal spacing of the *M* samples, in case these have been collected longitudinally. If such information is indeed available, it can be included in the model by treating the transformed CCF variables as functions of time, $\psi_{k}(t)$. On these functions, we impose a *Gaussian Process* prior (Gelman *et al.*, 2013; Rasmussen and Williams, 2006; Roberts *et al.*, 2013):

$$\psi_{k}(t)∼GP\left(0,\kappa (t,t\prime )\right)$$

where the kernel function κ(t,t′) encodes the covariance of $\psi_{k}(t)$ at times *t* and t′. This non-parametric approach permits modelling the time-dependency of the transformed CCF variables without any strong prior assumptions on the functional form of this dependency. The above implies that if *M* samples have been collected at times $t_{1}=0,\ldots ,t_{j},\ldots ,t_{M}=1$, then the variables $\psi_{jk}=\psi_{k}(t_{j})$ follow a multivariate Normal distribution:

$${\left\{\psi_{jk}\right\}}_{j=1}^{M}∼N_{M}(0_{M},K_{M})$$

where $0_{M}$ is the *M*-dimensional zero vector. The elements of the covariance matrix $K_{M}={\left\{\kappa (t_{j},t_{j^{\prime }})\right\}}_{j,j\prime }$ encode the covariance between the values of $\psi_{k}(t)$ at all possible pairs of sampling times *t_j_* and $t_{j^{\prime }}$.

We consider kernels of the form $\kappa (t,t\prime )=h^{2}g_{\tau }(t,t\prime ),$ where *h* is an amplitude parameter, while the function $g_{\tau }(t,t\prime )$, which is parameterized by an inverse squared time scale parameter *τ*, takes any of the following forms: (i) exponential: $g_{\tau }(t,t\prime )=e^{-\sqrt{\tau }|t-t^{\prime }|}$, (ii) Mat32: $g_{\tau }(t,t\prime )=(1+\sqrt{3\tau }|t-t\prime |)e^{-\sqrt{3\tau }|t-t|}$, (iii) Mat52: $g_{\tau }(t,t\prime )=(1+\sqrt{5\tau }|t-t\prime |+\frac{5{\left(t-t\right)}^{2}}{3\tau })e^{-\sqrt{5\tau }|t-t^{\prime }|}$ and (iv) exponentiated quadratic: $g_{\tau }(t,t\prime )=e^{-\tau {\left(t-t^{\prime }\right)}^{2}/2}$. These four kernels are members of the Matérn family of covariance functions ordered in terms of increasing smoothness (Rasmussen and Williams, 2006). Finally, we impose gamma priors on the amplitude and time scale parameters, $h^{2}∼G(1,1)$ and τ∼G(1,1).

### 2.4 Multi-output Gaussian process model for longitudinal tumour samples

In the above model, the cluster-specific scalar-valued functions $\psi_{k}(t)$ share the same Gaussian Process prior, but they are otherwise independent. We can directly model possible correlations between different clusters (i.e. different values of *k*) by assuming that the vector-valued function of time, $\psi (t)={\left\{\psi_{k}(t)\right\}}_{k=1}^{K}$, follows a Gaussian Process prior:

$$\psi (t)∼GP\left(0_{K},\lambda_{K}(t,t\prime )\right)$$

where $\lambda_{K}(t,t\prime )$ is a matrix-valued kernel encoding the *K *×* K* covariance matrix between vectors ψ(t) and ψ(t′). Given *M* longitudinally observed samples, the above implies that the matrix of CCF values $\Psi_{M\times K}={\left\{\psi_{jk}\right\}}_{j,k}$ follows a multivariate Normal distribution of dimensionality *MK*:

$$\text{vec}(\Psi_{M\times K})∼N_{MK}(\text{vec}(0_{M\times K}),\Lambda_{MK\times MK})$$

where the operator vec(·) vectorizes its matrix argument by stacking its columns on top of each other, $0_{M\times K}$ is a matrix of zeros and $\Lambda_{MK\times MK}$ is a positive semi-definite block matrix encoding the covariance between *ψ_jk_* and $\psi_{j\prime k\prime }$.

Assuming that the above kernel is *separable* (Álvarez *et al.*, 2012), we can write the factorization $\lambda_{K}(t,t\prime )=g_{\tau }(t,t\prime )\Sigma_{K}$, where $g_{\tau }(t,t\prime )$ is the same as in the previous section. $\Sigma_{K}$ is a positive semi-definite matrix factorized as $\Sigma_{K}=DCD$, where $D=\text{diag}(h_{1},\ldots ,h_{K})$ and $C\propto |C{|}^{\eta -1}$ is a correlation matrix following the LKJ prior (Stan Development Team, 2020) with concentration parameter *η*. A value of *η* = 1 implies a uniform prior over correlation matrices, while *η* = 2 (the value we adopt here) concentrates more probability mass around the identity matrix. This structure for $\Sigma_{K}$ implies both cluster-specific amplitudes $h_{k}^{2}$, as well as correlations between clusters. Alternatively, we can assume that $\Sigma_{K}=\text{diag}(h_{1}^{2},\ldots ,h_{K}^{2})$, which implies that different clusters have different values of the amplitude parameters $h_{k}^{2}$.

Finally, we examine the case where $\Lambda_{MK\times MK}$ is a block-diagonal matrix, with each of the *K* matrices along its main diagonal induced by the kernel $\kappa (t,t\prime )=h_{k}^{2}g_{\tau_{k}}(t,t\prime )$, where both amplitude $h_{k}^{2}$ and time scale *τ_k_* parameters are cluster-specific.

### 2.5 Relation between VAF and CCF

In this section, we give more details about the form of the function $\theta_{ij}=f({\tilde{\phi }}_{ij})$, which encodes the relationship between VAF and CCF of mutation *i* in sample *j*. With respect to mutation/locus *i*, each sample is viewed as a mixture of three cell populations (Roth *et al.*, 2014): (i) a normal population of $C_{j}^{N}$ non-cancer cells, (ii) a reference population of $C_{ij}^{R}$ cancer cells, which do not harbour mutation *i* and (iii) a variant population of $C_{ij}^{V}$ cancer cells, which harbour mutation *i*. The total number of cancer cells in the sample is $C_{j}^{T}=C_{ij}^{R}+C_{ij}^{V}$. The reference and variant populations may each be further subdivided into sub-populations, where a different number of chromosomes covers locus *i* in each sub-population. The total number of chromosomes in the normal, reference and variant populations overlapping locus *i* in sample *j* are, respectively, equal to $2C_{j}^{N}$ (assuming diploid normal cells), $D_{ij}^{R}C_{ij}^{R}$ and $D_{ij}^{V}C_{ij}^{V}$, where $D_{ij}^{R}$ and $D_{ij}^{V}$ are the average numbers of chromosomes per cell covering locus *i* in sample *j* in each of the two cancer cell populations. Similarly, the total number of chromosomes harbouring mutation *i* in sample *j* is equal to $d_{ij}^{V}C_{ij}^{V}$, where $d_{ij}^{V}$ is the *multiplicity*, i.e. the average number of chromosomes per cell in the variant cancer cell population harbouring mutation *i* in sample *j*. We write:

$$\begin{array}{c} \theta_{ij}=\frac{d_{ij}^{V}C_{ij}^{V}}{2C_{j}^{N}+D_{ij}^{R}C_{ij}^{R}+D_{ij}^{V}C_{ij}^{V}}\\ =\frac{d_{ij}^{V}\rho_{j}{\tilde{\phi }}_{ij}}{2(1-\rho_{j})+D_{ij}^{R}\rho_{j}(1-{\tilde{\phi }}_{ij})+D_{ij}^{V}\rho_{j}{\tilde{\phi }}_{ij}}\\ =f({\tilde{\phi }}_{ij}) \end{array}$$

where $\rho_{j}=C_{j}^{T}/(C_{j}^{N}+C_{j}^{T})$ is the purity of the tumour and ${\tilde{\phi }}_{ij}=C_{ij}^{V}/(C_{ij}^{R}+C_{ij}^{V})$. At this stage, two simplifying assumptions are often made: (i) there are no sub-clonal copy number events, which implies that $d_{ij}^{V},\;D_{ij}^{V}$ and $D_{ij}^{R}$ are whole numbers, and (ii) the reference and variant cancer cell populations have the same copy number profile at locus *i* in sample *j*, i.e. $D_{ij}^{R}=D_{ij}^{V}=D_{ij}$. Under these assumptions, the above expression simplifies to:

$$\begin{array}{c} \theta_{ij}=\frac{d_{ij}^{V}\rho_{j}}{2(1-\rho_{j})+D_{ij}\rho_{j}}{\tilde{\phi }}_{ij}\\ =\zeta_{ij}{\tilde{\phi }}_{ij} \end{array}$$

where *ζ_ij_* is the value of *θ_ij_* if mutation *i* in sample *j* is clonal (i.e. ${\tilde{\phi }}_{ij}=1$). The quantities *ρ_j_* and *D_ij_* can be independently estimated using software such as ASCAT (Van Loo *et al.*, 2010), ABSOLUTE (Carter *et al.*, 2012), TITAN (Ha *et al.*, 2014) and others, and they are considered fixed. One way to approximate the multiplicity $d_{ij}^{V}$ is as follows: first, we calculate $u_{ij}=d_{ij}^{V}{\tilde{\phi }}_{ij}=\theta_{ij}\rho_{j}^{-1}(2(1-\rho_{j})+D_{ij}\rho_{j})$. Then, we estimate $d_{ij}^{V}$ using the following rule:

$$d_{ij}^{V}=\left\{\begin{array}{ll} \left[u_{ij}\right] & u_{ij}\geq 1\\ 1 & u_{ij}<1 \end{array}\right.$$

where $\left[u_{ij}\right]$ is the closest integer to *u_ij_*. For a justification of this estimation procedure, see Dentro *et al.* (2017).

### 2.6 Observation models

We complete the above models by introducing expressions for the distribution of the read counts *r_ij_* harbouring mutation *i* in sample *j*. Since high-throughput sequencing data often exhibit over-dispersion, we consider a beta-binomial model:

$$r_{ij}∼\text{BBin}\left(R_{ij},v_{j}f({\tilde{\phi }}_{ij}),v_{j}(1-f({\tilde{\phi }}_{ij}))\right)\;\;\frac{1}{1+v_{j}}∼U(0,1)$$

where *R_ij_* is the sum of reads harbouring the alternative and reference alleles at locus *i* in sample *j* and *v_j_* is a precision parameter. In the absence of over-dispersion (i.e. when $v_{j}\to \infty$), the above reduces to the binomial model, $r_{ij}∼\text{Bin}\left(R_{ij},f({\tilde{\phi }}_{ij})\right)$. Both error models capture the discrete sampling of reads in the NGS data-generating process and they account for read depth variability due to non-uniform coverage across the genome. Parameter *v_j_* in the beta-binomial model is sample-specific, which allows the model to adapt to different degrees of over-dispersion across samples. Alternatively, a common precision parameter can be used for all samples in the absence of a sufficiently large volume of data. In Supplementary Methods, we further present a version of the above model, which explicitly accounts for possible sequencing errors.

### 2.7 Inference

We implemented the above models using the probabilistic programming language PyMC3 v3.8 (Salvatier *et al.*, 2016) and inference was conducted using Automatic Differentiation Variational Inference (ADVI; Kucukelbir *et al.*, 2017), instead of developing bespoke estimation algorithms, which is a rather laborious process particularly when multiple candidate models are considered (Vavoulis, 2020; Vavoulis *et al.*, 2015, 2017). Variational inference (VI; Blei *et al.*, 2017; Zhang *et al.*, 2019) is a computationally efficient approach for Bayesian inference, which aims to approximate the posterior density p(z|y) of latent variables **z** given data **y** using a surrogate probability density $q_{\eta }(z)$ parameterized by a vector of variational parameters η. In our case, the data **y** are the locus- and sample-specific read counts *r_ij_* and *R_ij_*, the local copy numbers *D_ij_*, the sample-specific purities *ρ_j_* and the sample collection times *t_j_*, while the latent variables **z** are the CCFs $\phi_{jk}$, the cluster weights *w_k_*, the amplitudes $h_{k}^{2}$, the time-scales *τ_k_* and the sample-specific dispersions *v_j_*. VI approximates p(z|y) by maximizing the lower bound of the marginal likelihood (or *evidence*) p(y), which is known as the *evidence lower bound* (ELBO), with respect to the variational parameters η:

$$p(y)\geq \underset{\text{ELBO}}{\underset{︸}{\overset{\text{entropy}}{\overset{︷}{H\left(q_{\eta }(z)\right)}}-\overset{\text{energy}}{\overset{︷}{\left(-\int q_{\eta }(z)p(y,z)\text{d}z\right)}}}}$$

Maximizing the ELBO is equivalent to jointly maximizing the entropy term (which leads to a more spread out variational distribution *q* and prevents over-fitting) and minimizing the average energy term (i.e. the discrepancy between *q* and *p*). Furthermore, the maximized ELBO, being a lower bound of the evidence p(y), can be used for model comparison (see below).

### 2.8 Performance metrics

We fit the above models against actual or simulated tumour samples (see Section 3). In the first case, the ground truth or *latent structure* of the data (i.e. the true CCFs, the number and composition of mutation clusters) is by definition unobservable and therefore unknown. This situation is the rule, not the exception, in the study of complex systems and it complicates model validation and selection, since model estimates cannot be verified against their true values. In this case, we compare the performance of different models using the model evidence, as approximated by the maximized ELBO (with a higher value indicating a better model). This criterion naturally favours simple models over complex ones, thus protecting against over-fitting (a manifestation of *Occam’s razor*). It is implied that models with higher ELBO better approximate the data generating process and, by extension, the underlying latent structure of the data. In the case of simulated data, the ground truth is known *a priori* and different models are compared using the *Adjusted Rand Index* (ARI), as implemented in the Python package scikit-learn v0.22 (Pedregosa *et al.*, 2011). ARI takes values between -1 and 1, with negative or close to 0 values indicating deviation from the ground truth, while values close to 1 indicate close agreement to it. ARI is symmetric, and for this reason, we also use it for estimating the concordance between any two clustering models when these are fitted on actual data. In Supplementary Methods, we give further details on ARI and on two additional metrics, the *Adjusted Mutual Information* (AMI) and the *Fowlkes-Mallows Index* (FMI). All three scores are robust against agreement-by-chance and anisotropic cluster shapes.

### 2.9 Model nomenclature

In Section 3, the various models described above are referred to as follows. The model that assumes a uniform (i.e. flat) prior over the CCF variables $\phi_{jk}$ is the **Flat** model. The model that assumes a single-output Gaussian Process prior over the transformed CCF variables *ψ_jk_* is the **GP0** model. The models assuming a multi-output Gaussian Process prior on *ψ_jk_* are labelled **GP1** (when $\Sigma_{K}$ is diagonal), **GP2** (when $\Sigma_{K}$ is full rank) and **GP3** (when $\Lambda_{MK\times MK}$ is block-diagonal with cluster-specific $h_{k}^{2}$ and *τ_k_* parameters), respectively. Each of the models **GP0** to **GP3** admits exponential (**Exp**), **Mat32**, **Mat52** or exponentiated quadratic (**ExpQ**) kernels and are labelled accordingly, e.g. **GP0-Exp**, **GP0-ExpQ**, etc. In total, we examined 17 models. If the number of parameters in the **Flat** model is $n_{p}=L+M\cdot L$ (where *L* is the number of clusters with non-zero weights), the number of parameters in the **GP0** to **GP3** models is $n_{p}+2,\;n_{p}+L+1$, $n_{p}+L+1+L(L-1)/2$ and $n_{p}+2L$, respectively.

## 3 Results

We conducted a series of computational experiments on WES and WGS data from patients with CLL (González-Rincón *et al.*, 2019; Schuh *et al.*, 2012), on TGS data from the liquid biopsy of a patient with melanoma (Cutts *et al.*, 2017) and on simulated data. The aim of these experiments was to demonstrate the application of the above models on longitudinal data and to assess their relative performance.

### 3.1 The case of patient CLL003

**Fig. 1.**
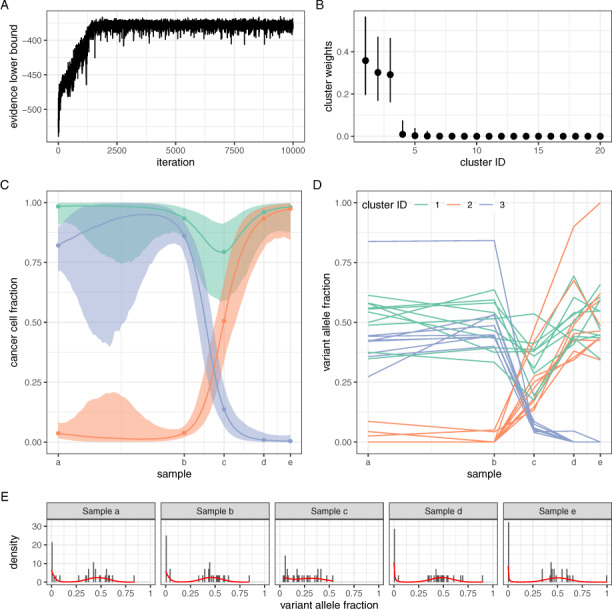
Application of model **GP0-Mat32** on data from patient CLL003 (Schuh *et al.*, 2012). (**A**) Parameter estimation was achieved via maximization of the evidence lower bound. Convergence was attained in less than 3K iterations. (**B**) The number of clusters in the data was automatically estimated through the use of a Dirichlet Process prior. In this example, three major clusters were identified. (**C**) The temporal profile of the three major clusters during disease treatment and progression. The median and 95% credible intervals are shown. Sample collection took place over the course of 35 months. (**D**) Observed VAF values for each somatic mutation and their cluster assignment. (**E**) The fitted model (red lines) against the data in each sample

**Fig. 2.**
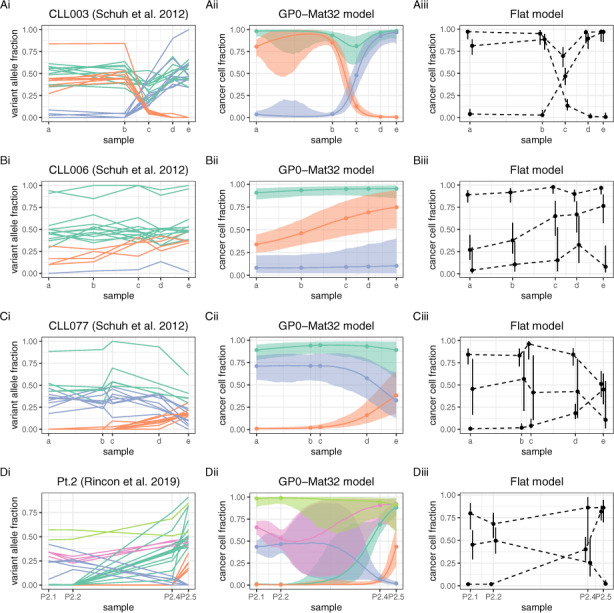
Overview of CLL data and fitted models **Flat** and **GP0-Mat32**. Unlike **GP0-Mat32**, the **Flat** model estimates the CCF of each cluster only at the points of sample collection (dashed lines). Although both models identified the same number of clusters in datasets CLL003 to CLL077, these were not concordant (see main text for details)

**Fig. 3.**
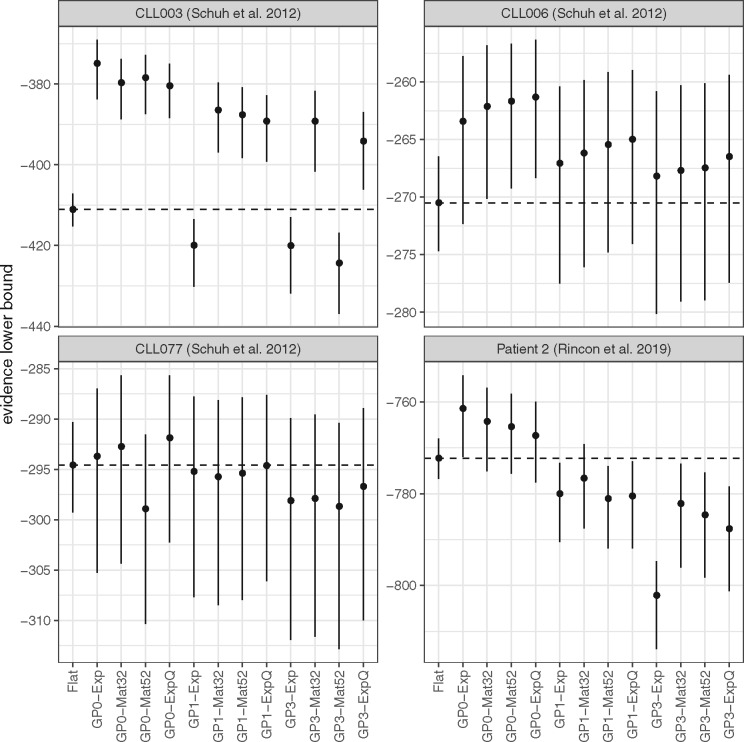
Comparison of all models using the data in the previous figure and the evidence lower bound for assessing performance. **GP0** models perform at least as well as the **Flat** model in all cases. Models **GP2** had the worst performance and they were omitted from the figure

First, we demonstrate the application of model **GP0-Mat32** on WGS data from patient CLL003 reported by Schuh *et al.* (2012) (Fig. 1; the performance of other models on the same dataset is summarized in Figures 2A and 3, top-left panel; see also Supplementary Figs S1–S3). Details on sequencing and bioinformatics analysis for obtaining this data are given in the original paper. Briefly, peripheral blood was collected at five specific time points during disease progression, treatment and relapse together with a matched buccal swab (for germinal DNA). All samples underwent WGS followed by bioinformatics analysis, which identified 28 somatic mutations. Fitting the model to this data was performed by maximizing the ELBO (see Section 2), which can be used for assessing convergence of the estimation algorithm (typically achieved in less than 3K iterations; Fig. 1A). Following a non-parametric approach for clustering mutations using a Dirichlet Process prior on the CCFs (see Section 2) means that the number of clusters is not selected *a priori*, but rather estimated along with other model parameters (Fig. 1B). We identified three major mutation clusters: one with median weight ≈35% (i.e. any mutation has approximately 35% probability of belonging to this cluster) and two slightly smaller clusters with median ≈30%. In Figure 1C, we illustrate the evolution of each cluster in time. Sample (a) was collected before commencing treatment with chlorambucil; sample (b) before treatment with fludarabine, cyclophosphamide and rituximab (FCR); sample (c) immediately after six cycles of FCR; sample (d) before treatment with ofatumumab; and sample (e) after treatment with ofatumumab, spanning in total a period of 35 months. Initial treatment with chlorambucil did not alter significantly the prevalence of the three mutation clusters, with median CCF > 75% for clusters 1 and 3 and median CCF < 10% for cluster 2. The second treatment regime (FCR) induced a dramatic reduction in the prevalence of cluster 3, but only a minor reduction of cluster 1. Concomitantly, the prevalence of cluster 2 increased substantially. By the end of the 35-months period, cluster 1 had recovered and, along with cluster 2, it reached CCF values higher than 95%, while cluster 3 collapsed. Our algorithm soft-clusters mutations, i.e. for each mutation, it calculates the probability of membership to each cluster. From these, a hard clustering can be obtained by assigning each mutation to the cluster with the highest median membership probability. Figure 1D illustrates the hard cluster assignment for each mutation in the CLL003 dataset. It is interesting to observe that, by considering multiple time-separated samples, our method manages to deconvolve mutation clusters with similar VAF values, which would otherwise be hard to distinguish [e.g. observe the mixing of clusters 1 and 3 at time points (a) and (b) or clusters 1 and 2 at time points (d) and (e)]. Finally, we can visually confirm the goodness of fit of the model to the data by overlaying the posterior predictive distribution (red lines in Fig. 1E) on the histograms of observed VAF values for each sample.

### 3.2 Benchmarks on CLL data with four or five samples

Next, we applied the remaining models on the data from patient CLL003, as well as all models on data from patients CL006 and CLL077 reported in Schuh *et al.* (2012) (models **Flat** and **GP0-Mat32** are illustrated in Fig. 2; the performance of all models, except **GP2**, is summarized in Fig. 3). WGS and bioinformatics analysis were conducted as for patient CLL003 (see original paper for details). For patients CLL006 and CLL077, samples were collected over a period of 50 and 57 months, respectively. In addition, we examined WES data from Patient 2 reported in the study by González-Rincón *et al.* (2019), where collected samples spanned 79 months in total (for details of sequencing and bioinformatics analysis, see original paper).

There were 18, 21 and 32 somatic mutations in patients CLL006, CLL077 and Patient 2, respectively (as well as 28 somatic mutations in patient CLL003, as previously mentioned; Fig. 2Ai–Di). A preliminary comparison indicates that, for patients CLL003 to CLL077, model **GP0-Mat32** (Figs. 2Aii–Cii) identified the same number of mutation clusters as the simpler **Flat** model (Fig. 2Aiii–Ciii), i.e. three clusters with similar temporal dynamics. To assess the clustering concordance between the two models (i.e. whether they assign the same mutations to the same clusters), we calculated the values of ARI, which were equal to 0.54, 0.79 and 0.58, respectively. This indicates that the two models are not perfectly concordant in any of these three datasets (despite both identifying the same number of clusters) presumably due to the partial overlap between different mutation groups, as illustrated in Figure 2Ai–Ci. One striking difference between the **Flat** and **GP**-based models is that while the former estimates the latent state of the tumour only at the time points of sample collection (this is indicated by the dashed connecting lines in Fig. 2Aiii–Ciii), the latter provides an estimate of the complete history of this latent state, i.e. both at and between these fixed time points. This is a major difference in favour of the use of **GP**-based models. In the case of Patient 2, the **Flat** and **GP0-Mat32** models identify three and five clusters, respectively (ARI = 0.63; Fig. 2Di–iii). For comparison, in the original paper, the authors identified seven clusters using PyClone (Roth *et al.*, 2014).

To further assess the relative performance of different models (and without knowledge of the true clonal state of each tumour), we used the ELBO as performance metric (see Section 2). The ELBO provides a lower bound on the marginal likelihood of the data (i.e. the evidence) and, at the same time, it includes an internal mechanism that prevents over-fitting. Thus, it is often used in practise for model comparison and selection, with higher ELBO values indicating a better model. As illustrated in Figure 3A, all **GP0** models, all but one **GP1** models and all but two **GP3** models outperform the **Flat** model on the CLL003 data. The **GP2** models, which have the largest number of parameters, were by far the worst performers on these datasets and they are omitted from the figure. There is a clear trend of decreasing performance with increasing number of parameters among the **GP**-based models, which is not surprising given that the lower the number of time points, the lower the capacity of the data to support overly complex models (as, for example, in the case of **GP2** models). In the case of CLL006 (Fig. 3B), the same trend is observed, although the difference of the **GP**-based models from the **Flat** model is less pronounced. In the case of CLL077 (Fig. 3C), models **GP0-Mat32** and **GP0-ExpQ** perform better than the **Flat** model (although this difference is not particularly pronounced because of the high variance of the ELBO), but the remaining **GP**-based models perform either clearly worse or comparably to the **Flat** model. In the case of Patient 2 (Fig. 3D), the **GP0** models are again the best performers, unlike **GP1**and **GP3** models, which are clearly worse than the **Flat** model. In summary, there is always at least one member of the relatively parsimonious (in terms of the number of model parameters) **GP0** family of models that performs better than the **Flat** model in the above benchmarks.

### 3.3 Benchmarks on CLL and melanoma data with 10 or 13 samples

**Fig. 4.**
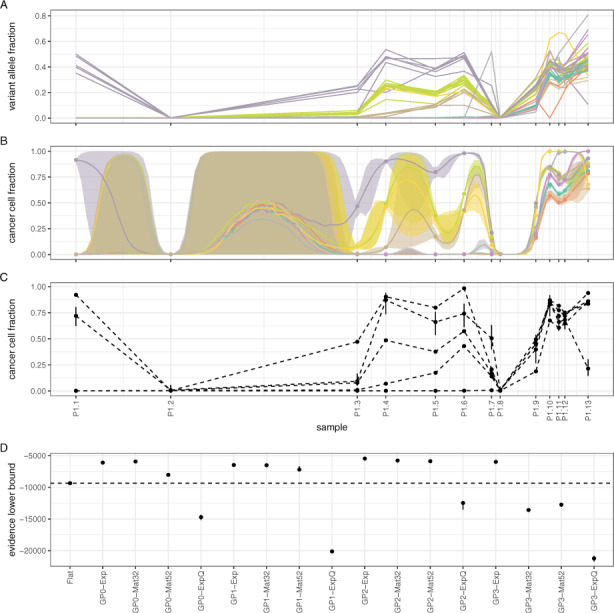
Assessing model performance on CLL data from Patient 1 (González-Rincón *et al.*, 2019). (**A**) Observed VAF values for each somatic mutation over 6.5 years and their cluster assignments (colours are the same as in (**B**). B) Mutation clusters identified by model **GP0-Mat32**. (**C**) Mutation clusters identified by the **Flat** model. (**D**) Comparative performance of various models. Notice that simpler models (**GP0**) often perform equivalently to or better than more complex ones (**GP1**, **GP2** and **GP3**)

Next, we tested our models on longitudinal genomic data involving a higher number of time points. The first dataset comes from Patient 1 in González-Rincón *et al.* (2019). A total of 13 peripheral blood mononuclear cell samples (P1.1–P1.13) were collected over the course of 6.5 years and underwent TGS. Samples were collected before or after treatment commenced. In particular, sample P1.1 was collected before the patient received a stem cell transplant and the same holds for sample P1.8. Bioinformatics analysis identified 46 somatic mutations over all 13 samples (Fig. 4A; see original paper for details). Model **GP0-Mat32** identified nine mutation clusters (Fig. 4B), while the **Flat** model identified five (Fig. 4C). For comparison, in the original paper, the authors estimated four clusters using PyClone (Roth *et al.*, 2014). Overall, models **GP0**, **GP1** and **GP2** perform better than the **Flat** model, unless an exponentiated quadratic kernel (**ExpQ**) is used (Fig. 4D). We speculate that this is because **ExpQ** encodes perfectly smooth dynamics, which presumably cannot model sufficiently well the non-smooth bottleneck points P1.2 and P1.8 which precede stem cell transplantation. Model **GP3-Exp** is also performing better than the **Flat** model.

**Fig. 5.**
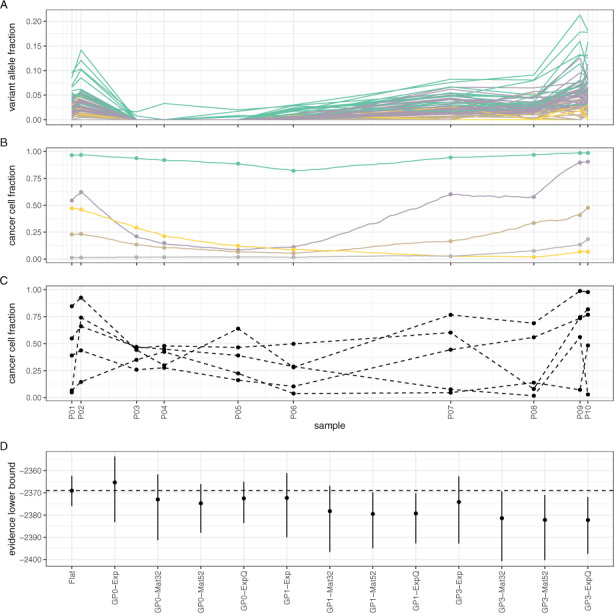
Assessing model performance using data from a liquid biopsy on a subject with melanoma (Cutts *et al.*, 2017). (**A**) Observed VAF values for each somatic mutation over 13 months of treatment and their cluster assignments (colours are the same as in **B**). B) Mutation clusters identified by model **GP0-Exp** (due to extensive overlap, credible intervals are omitted for clarity). (**C**) Mutation clusters identified by the **Flat** model. (**D**) Comparative performance of various models. Model **GP0-Exp** performs comparably to **Flat**

The second multi-sample dataset comes from the liquid biopsy of a patient with metastatic melanoma (Cutts *et al.*, 2017). Peripheral blood samples were collected at 10 different time points during pre-treatment, post-treatment and relapse over the course of 13 months. Targeted sequencing was conducted on extracted cell-free DNA followed by bioinformatics analysis, which revealed 63 somatic mutations. Visual inspection of the data indicates the absence of a definitive cluster structure (Fig. 5A) and, for this reason, this is an interesting dataset to use for model evaluation. Both the **Flat** and **GP0-Exp** models identified five mutation clusters with little concordance between them (ARI = 0.27) due to the extended overlap between different mutations bundles (Fig. 5B, C). The median performance of model **GP0-Exp** is nominally higher than the **Flat** model, although it is doubtful whether the difference is substantial due to the high variance of the ELBO (Fig. 5D). The remaining **GP**-based models perform worse than either **Flat** or **GP0-Exp**.

### 3.4 Computational experiments on simulated data

Overall, models **GP0** (particularly **GP0-Exp** and **GP0-Mat32**) perform at least as well as the **Flat** model in all the above datasets. More complex models (i.e. models with a larger number of parameters), such as **GP1**, **GP2** and **GP3**, require a higher number of longitudinally collected samples for improved performance (Fig. 4). However, this is not a sufficient condition, since data of low complexity (i.e. with trivial or non-obvious cluster structure and dynamics) can negatively affect the performance of the **GP**-based models (Fig. 5).

We wanted to test whether these trends (i.e. the reduction in the performance of the **GP**-based models in relation to the **Flat** model as data size and complexity decreases) can be replicated using synthetic genomic data. For a given number of samples *M*, mutations *N* and mutation clusters *K*, data were simulated using actual experimental data as template (see Supplementary Methods for details). In total, we generated 729 datasets, each of which was processed using the **Flat** and **GP0** models (which were top performers on the actual data) and their performance was assessed against the true cluster structure of the dataset.

**Fig. 6.**
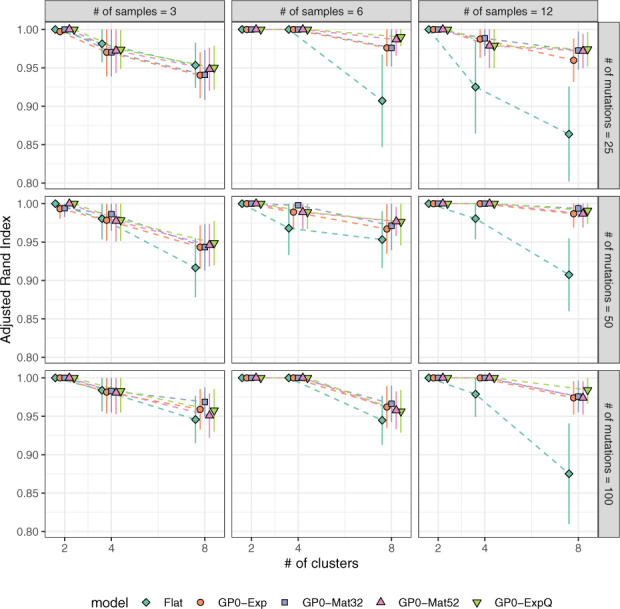
Benchmarks on synthetic data. When the number of samples is small (*M *=* *3) or data complexity (i.e. the number of clusters) is low (*K *=* *2), the **Flat** model performs comparably to the **GP0** models. In all other cases, it is outperformed by them. Parameters used in data simulation were informed by the experimental data (see main text for details)

We may observe that when few samples are available (*M *=* *3), the baseline model (**Flat**) performs comparably to **GP0** at all values of *N* and *K* (Fig. 6). For large (*M *=* *12) datasets, the **Flat** model falls behind the other models, when the number of clusters in the data is relatively high (*K *=* *4 or 8). At medium sample numbers (*M *=* *6), the same effect is observed at small mutation numbers (*N *=* *25). These results indicate that in the presence of non-trivial cluster dynamics, the baseline model is comparable to **GP0** models, but only when the number of samples or data complexity (here, the number of clusters) is low. In Supplementary Results, we provide further benchmarks against PyClone (Roth *et al.*, 2014) and Canopy (Jiang *et al.*, 2016), as well as additional performance metrics (Supplementary Figs S4–S6).

## 4 Discussion

Tumour heterogeneity in the form of distinct cancer cell populations or clones is the outcome of a process of continuous adaptation of the component cells to their local micro-environment. The outcome of any therapeutic intervention depends on this latent cellular diversity and, for this reason, statistical methodologies that help deconvolve the clonal structure of tumours are valuable tools at the disposal of clinicians and bioinformaticians.

Building on previous works (Nik-Zainal *et al.*, 2012; Roth *et al.*, 2014), we propose a statistical methodology for clonal deconvolution based on longitudinal data, which explicitly considers the temporal spacing of sample collection. Our approach combines two Bayesian non-parametric statistical frameworks, namely Dirichlet Process Mixture Models (for clustering in the absence of prior knowledge on the number of clusters supported by the data) and Gaussian Process Latent Variable Models (for modelling the time-dependence of clone prevalence without any explicit assumptions on the form of this dependence). The models we present in this article are sufficiently flexible to capture many common scenarios without additional assumptions or constraints, including monotonic increase and/or decrease of CCFs, as well as stability of CCFs near (or at) 0 or 1. More elaborate scenarios (e.g. the introduction of change-points in the function domain) are possible through appropriate design of the kernel function, possibly at the cost of increased model complexity.

Using a combination of experimental data from patients with CLL or melanoma, as well as synthetic data simulated using experimental data as template, we demonstrate that there are advantages in this approach, when compared to several baseline models (Fig. 6 and Supplementary Figs S4–S6). These benefits are particularly evident when longitudinal data of sufficient volume and complexity are available. When this is not the case, our methodology performs comparably to baseline models, but it also manages to reconstruct the time dependence of mutation clusters continuously in time (i.e. not only at the points of sample collection, which is what baseline models do, but also between them) from a small number of sequentially collected samples.

CLL is an ideal experimental model for the study of cancer evolution, because it develops over many years and because the collection of a long sequence of blood samples from the same patient for genomic analysis is easy, at least when compared to solid tumours. Thus, we expect that our methodology will find applications in the study of CLL and other liquid cancers. It can also be used as a general purpose clustering tool for identifying populations of mutations based on sequencing of circulating tumour DNA obtained through a liquid biopsy.

As with other approaches for clustering mutations based on bulk sequencing data, a phylogeny is not derived directly, but it can be calculated retrospectively using the output of our method as input to bespoke software (Dang *et al.*, 2017; Niknafs *et al.*, 2015; Qiao *et al.*, 2014). Furthermore, single-cell sequencing promises to alleviate the confounding of clones inherent in methods based on bulk sequencing by permitting direct observation of the genotypes of the cells that compose each clone. However, it is in turn plagued by its own technical limitations, namely high levels of noise, error rates and missing values (Borgsmueller *et al.*, 2020; Chen *et al.*, 2019; El-Kebir, 2018; Jahn *et al.*, 2016; Malikic *et al.*, 2019; Ramazzotti *et al.*, 2020; Ross and Markowetz, 2016; Roth *et al.*, 2016; Zafar *et al.*, 2017, 2019).

Finally, an important assumption in our approach is the absence of sub-clonal copy number variation [see assumptions (a) and (b) in Section 5]. Although commonly adopted by statistical methods of sub-clonal reconstruction based on single nucleotide variants (SNV), we recognize that these assumptions may not always be exact. It is possible to relax these assumptions, however, this would lead to an intractable estimation problem in the resulting model. A possible mitigation strategy is to complement a SNV-based approach as the one we present in this article (which, as we show, does incorporate a correction for copy number variation, CNV) by subsequently applying a CNV-based approach as well. Several such methods are available aiming to identify the copy number state of each sub-clone in a tumour by typically using the measured *B-allele frequency* as input to downstream estimation procedures (Carter *et al.*, 2012; Fischer *et al.*, 2014; Ha *et al.*, 2014; Nik-Zainal *et al.*, 2012).

## 5 Conclusion

In conclusion, we propose that considering information on the temporal spacing of longitudinal tumour samples can improve clonal deconvolution and we show how this can be achieved in the context of non-parametric Bayesian statistics.

## Supplementary Material

btaa672_Supplementary_Data
