# Supplementary material for: A statistical approach for tracking clonal dynamics in cancer using longitudinal next-generation sequencing data
Source: Bioinformatics. 2020 Jul 28;37(2):147–54. doi: 10.1093/bioinformatics/btaa672 (PMC8055230; doi:10.1093/bioinformatics/btaa672)
Supplement: btaa672_Supplementary_Data [file btaa672_supplementary_data.pdf]

Supplementary material: A statistical approach for tracking clonal dynamics in cancer using longitudinal next-generation sequencing data

Dimitrios V. Vavoulis[1,2,3,4,*], Anthony Cutts[1,4], Jenny C. Taylor[2,3] & Anna Schuh[1,3,4,5]

May 11, 2020

1. Department of Oncology, University of Oxford, Oxford, UK; 2. Wellcome Centre for Human Genetics, University of Oxford, Oxford, UK; 3. NIHR Oxford Biomedical Research Centre, University of Oxford, Oxford, UK; 4. Molecular Diagnostic Centre, Department of Oncology, University of Oxford, Oxford, UK; 5. Department of Haematology, Oxford University Hospitals NHS Trust, Oxford, UK

*dimitris.vavoulis@oncology.ox.ac.uk

1 Supplementary Methods

1.1 Model justification

In total, we examined 17 models in this paper. We consider the first (**Flat**) model as the *standard model*, since it encapsulates the major aspects of a large number of statistical models for clonal de-convolution. It does not explicitly include any temporal information and it is used as the baseline model. The remaining models explicitly incorporate temporal information through the use of Gaussian Process priors and they fall into four categories: **GP0**, **GP1**, **GP2** and **GP3**. The biological motivation behind these models is the following: it is natural to assume that the abundance of each mutational cluster (and each clone) is a function of time and since different clones compete for limited resources, all such functions in the same tumour are expected to be correlated. Models **GP0** (single-output Gaussian Process models) and **GP1** to **GP3** (multi-output Gaussian Process models) reflect different ways to model such correlations. Also, they correspond to different configurations of biologically meaningful properties of the aforementioned functions, such as their amplitudes (i.e. how large are the fluctuations in the abundances of mutation clusters?) and time scales (how rapidly do these fluctuations take place?). A final detail is how smooth these fluctuations are. For each **GP** model, the smoothness of cluster abundances as functions of time is modelled using one of four different kernels: **Exp**, **Mat32**, **Mat52** and **ExpQ**, which range from non-smooth/non-differentiable (**Exp**) to perfectly smooth/infinitely differentiable (**ExpQ**) functions. Different kernels potentially give rise to different temporal profiles for each mutation cluster.

1.2 Accounting for sequencing errors

It is not unusal for NGS technologies to introduce errors, which manifest as low-frequency artefacts in the generated sequencing data. A common strategy for handling these errors is to filter the data before further downstream analyses. For example, after initial screening of somatic variation data, the investigator may decide that variants with low VAF values (e.g. less than 1%) are noise and proceed to remove them from the dataset. Alternatively, such sequencing errors may be handled directly by appropriate modifications of the Beta-Binomial noise model. Assuming a small sequencing error rate ϵ, the beta-binomial model can be writen as $r_{ij} \sim \text{BBin}(R_{ij}, v_j\tilde{g}_{ij}, u_j(1-\tilde{g}_{ij}))$, where $\tilde{g}_{ij} = \epsilon + (1-\epsilon)f(\tilde{\phi}_{ij})$. In the absence of overdispersion ($v_j \to \infty$), the above reduces to the binomial model: $r_{ij} \sim \text{Bin}(R_{ij}, \epsilon + (1-\epsilon)f(\tilde{\phi}_{ij}))$. Under this formulation, the expected value of VAF is modelled as the mixture of two factors: tumour heterogeneity, which is summarised by the function $f(\tilde{\phi}_{ij})$, and the sequencing error ϵ. When the error rate is small, $\epsilon \ll f(\tilde{\phi}_{ij})$, the above two models reduce to the forms presented in the main text.

1.3 Performance metrics

In our benchmarks on synthetic data, we have used three different performance metrics: the *Adjusted Rand Index* (ARI; [1]), the *Adjusted Mutual Information* (AMI; [2]) and the *Fowlkes-Mallows Index* (FMI; [3]). All three scores are robust against agreement-by-chance between any two clusterings and against anisotropic cluster shapes. Below, we give details on how these metrics are calculated.

Given a set of N mutations and two independent clusterings of these mutations $X = (X_1, \ldots, X_k)$ and $Y = (Y_1, \ldots, Y_l)$, we can construct the following contingency table:

	Y_1	Y_2	$\ldots$	Y_l	$\sum_r$
X_1	n_{11}	n_{12}	$\ldots$	n_{1l}	a_1
X_2	n_{21}	n_{22}	$\ldots$	n_{2l}	a_2
$\vdots$	$\vdots$	$\vdots$	$\ddots$	$\vdots$	$\vdots$
X_k	n_{k1}	n_{k2}	$\ldots$	n_{kl}	a_k
$\sum_c$	b_1	b_2	$\ldots$	b_l	N

where n_{ij} is the number of mutations in common between X_i and Y_j, and a_i and b_j are row- and column-wise sums, respectively. Then, ARI is given by the following expression:

$$ARI = \frac{\sum_{ij}\binom{n_{ij}}{2} - \left[\sum_i \binom{a_i}{2}\sum_j \binom{b_j}{2}\right]/\binom{N}{2}}{\frac{1}{2}\left[\sum_i \binom{a_i}{2} + \sum_j \binom{b_j}{2}\right] - \left[\sum_i \binom{a_i}{2}\sum_j \binom{b_j}{2}\right]/\binom{N}{2}}$$

where $\binom{c}{d}$ indicates the *binomial coefficient*. ARI takes values between -1 and 1, where negative or close to 0 values indicate poor agreement between the two clusterings.

Next, the calculation of AMI requires first calculating the *Mutual Information* (MI) between clusterings X and Y, which is defined as follows:

$$MI = \sum_i^k \sum_j^l \pi_{ij} \log \frac{\pi_{ij}}{p_i q_j}$$

where $p_i = a_i/N$, $q_j = b_j/N$ and $\pi_{ij} = n_{ij}/N$. Then, the AMI is calculated as:

$$AMI = \frac{MI - \mathbb{E}[MI]}{\max(H_X, H_Y) - \mathbb{E}[MI]}$$

where $H_X = -\sum_i p_i \log p_i$ and $H_Y = -\sum_j q_j \log q_j$ are the entropies of X and Y, respectively. The expected MI (assuming X and Y are random) is given by the following expression:

$$\mathbb{E}[MI] = \sum_i^k \sum_j^l \sum_{\max(1, a_i+b_j-N)}^{\min(a_i,b_j)} \frac{n_{ij}}{N} \log\left(\frac{N \cdot n_{ij}}{a_i b_j}\right) \frac{a_i!\, b_j!\, (N-a_i)!\, (N-b_j)!}{N!\, n_{ij}!\, (a_i - n_{ij})!\, (b_j - n_{ij})!\, (N - a_i - b_j + n_{ij})!}$$

Values of AMI close to 0/1 indicate poor/excellent agreement between clusterings X and Y.

Finally, the FMI can be calculated easily as the geometric mean of the pairwise precision and recall, using the following expression:

$$FMI = \frac{TP}{\sqrt{(TP+FP)(TP+FN)}}$$

where TP is the number of pairs of mutations that belong in the same cluster in both X and Y; FP is the number of pairs that belong in the same cluster in X, but not in Y; and FN is the number of pairs that belong in the same cluster in Y, but not in X. As with the AMI, values of FMI close to 0/1 indicate poor/excellent agreement between X and Y.

1.4 Generation of synthetic data

For a given number of samples M, mutations N and mutation clusters K, data are simulated as follows: a) for each sample j, we randomly choose a purity value ρ_j between 80% and 90% and a random collection time t_j (with the first sample collected at time 0 and the last at time 1); b) for each cluster k, we sample a set of values $\{\psi_{jk}\}_{j,k}$ from a Gaussian process prior with squared amplitude h^2 and inverse squared time scale τ; we calculate each ϕ_{jk} as a sigmoid function of ψ_{jk}; c) for each mutation i, we randomly sample a cluster membership indicator z_i between 1 and K; d) finally, for each mutation i in each sample j, we sample the total number of reads R_{ij} from the empirical distribution of total reads in the data and then the number of mutated reads r_{ij} from a Binomial distribution: $r_{ij} \sim \text{Bin}(R_{ij}, \frac{1}{2}\rho_j \phi_{jz_i})$. We generated data with $M = \{3, 6, 12\}$, $N = \{25, 50, 100\}$ and $K = \{2, 4, 8\}$. For h^2 and τ, we used the values $\{1, 10, 20\}$ and $\{1, 10, 100\}$, respectively, which cover the range of values estimated from the actual data presented in the main text. For each of the 243 combinations of these parameters, we generated 3 replicates, which leads to a total of 729 datasets.

2 Supplementary results

In the main text, we used synthetic data to compare the performance of the baseline (i.e. **Flat**) model against a group of **GP0** models, which (unlike the **Flat** model) explicitly take into account information on the temporal spacing of longitudinally collected samples (see Fig. 6 in main text). Here, we extend these simulations by considering two additonal baseline models: **PyClone** [4] and **Canopy** [5]. **PyClone** is a well known software for clustering mutations with similar VAF values in one or more tumour samples. **Canopy** is also a popular software for clonal and phylogenetic tree reconstruction, which also provides facilities for clustering the mutational profiles of tumour samples. Both models were applied on exactly the same data as those presented in Fig. 6 in the main text.

Our results from this set of benhmarks are illustrated in Suppl. Fig. 4. When few samples are available ($M = 3$), the **Flat** and **PyClone** models perform similarly to the **GP0** models. At a high number of samples ($M = 12$), the performance of the baseline models (**Flat**, **PyClone**, **Canopy**) falls behind models **GP0** and this difference is more pronounced at low mutation numbers ($N = 25$ or 50) and high data complexity (i.e. high number of clusters, $K = 4$ or 8). At intermediate sample numbers ($M = 6$), the baseline models perform worse than the **GP0** ones when few mutations ($N = 25$) and many clusters ($K = 8$) are considered, but again this gap in performance closes with increasing mutation numbers. **Canopy** tends to perform worse that the alteratives, although all models demonstrate very high (i.e. above 85%) agreement with the ground truth in almost all examined scenarios. As stated in the main text, these results indicate that, in the presence of non-trivial cluster dynamics, the baseline models are comparable to **GP0** models, but only when the number of samples or data complexity (here, the number of clusters) is low. As illustrated in Suppl. Figs. 5 and 6, the same conclusions hold when agreement to the ground truth is measured using the *Adjusted Mutual Information* (AMI) or the *Fowlkes-Mallows Index* (FMI).

3 Supplementary Discussion

In the section *Model nomenclature* in the main text, we give the total number of parameters that need to be estimated in each model. In comparison to **Flat**, models in the **GP0** group (which are the ones performing best in our becnhmarks) inlcude only two additional parameters, while models **GP2** have $L + 1 + L(L - 1)/2$ more parameters (L is the number of clusters with non-zero weights) making them the most complex models we examined in this paper. The total number of data points considered for parameter estimation is $N \times M$, where M is the number of samples and N is the number of mutations (thus, for patient CLL003 with 28 mutations, we have 140 datapoints in total). The smallest dataset we studied was from patient CLL006 (18 mutations; 5 samples; therefore, 90 datapoints; Figs. 2Bii and Fig. 3, top-right panel). Models **GP0** perform quite well on this dataset, but models **GP2** had quite bad performance (both on this dataset and on the other datasets presented in the same figure) and, therefore, they were omitted from the figure. At larger datasets (e.g. Fig. 4D), most **GP2** models perform quite well (i.e. better than the **Flat** baseline), but comparably to the simpler **GP0** models. Overall, the **GP0** group of models perform at least as well as (and usually better than) the baseline **Flat** model on the datasets we analysed. In the case of synthetic data, models **GP0** perform comparably to the baseline in the case of 25 mutations and 3 samples (75 data points; Fig. 6, top-left panel and Suppl. Figs 4 to 6, top-left panel) and their performance gap (compared to the baseline) increases with increasing number of samples and data complexity.

Below we give some guidance on when to use each model. We recommend using the **Flat** model in the case of single tumours or cross-sectional samples and one of the **GP0** models or the **Flat** model in the case of longitudinal data. In order to decide which model to use in this case, the user is advised to run a small benchmark on their longitudinal dataset comparing the **Flat** against the four **GP0** models using the ELBO as performance metric. If **Flat** is the best performing model, then the user should use this model. If one of the **GP0** models performs at least as well as the **Flat** model, then the user is advised to use the **GP0** model, instead. If more than one **GP0** models perform at least as well as the **Flat** model, but they have similar performance to each other, then the user is advised to use the smoothest model, because smoother models estimate more narrow 95% credible intervals (see Fig. 1 and Suppl. Figs. 1 to 3). Notice that the **GP0** models are ordered as **GP0-Exp** $<$ **GP0-Mat32** $<$ **GP0-Mat52** $<$ **GP0-ExpQ** in terms of increasing smoothness, where **GP0-Exp** is not smooth (i.e. non-differentiable), while **GP0-ExpQ** is perfectly smooth (i.e. infinitely differentiable).

References

[1] Lawrence Hubert and Phipps Arabie. Comparing partitions. *J. Classification*, 2(1):193–218, December 1985.

[2] Nguyen Xuan Vinh, Julien Epps, and James Bailey. Information theoretic measures for clusterings comparison: Variants, properties, normalization and correction for chance. *J. Mach. Learn. Res.*, 11(95):2837–2854, 2010.

[3] E B Fowlkes and C L Mallows. A method for comparing two hierarchical clusterings. *J. Am. Stat. Assoc.*, 78(383):553–569, September 1983.

[4] Andrew Roth, Jaswinder Khattra, Damian Yap, Adrian Wan, Emma Laks, Justina Biele, Gavin Ha, Samuel Aparicio, Alexandre Bouchard-Côté, and Sohrab P Shah. PyClone: statistical inference of clonal population structure in cancer. *Nat. Methods*, 11(4):396–398, April 2014.

[5] Yuchao Jiang, Yu Qiu, Andy J Minn, and Nancy R Zhang. Assessing intratumor heterogeneity and tracking longitudinal and spatial clonal evolutionary history by next-generation sequencing. *Proc. Natl. Acad. Sci. U. S. A.*, 113(37):E5528–37, September 2016.

[6] Anna Schuh, Jennifer Becq, Sean Humphray, Adrian Alexa, Adam Burns, Ruth Clifford, Stephan M Feller, Russell Grocock, Shirley Henderson, Irina Khrebtukova, Zoya Kingsbury, Shujun Luo, David McBride, Lisa Murray, Toshi Menju, Adele Timbs, Mark Ross, Jenny Taylor, and David Bentley. Monitoring chronic lymphocytic leukemia progression by whole genome sequencing reveals heterogeneous clonal evolution patterns. *Blood*, 120(20):4191–4196, November 2012.

Figures

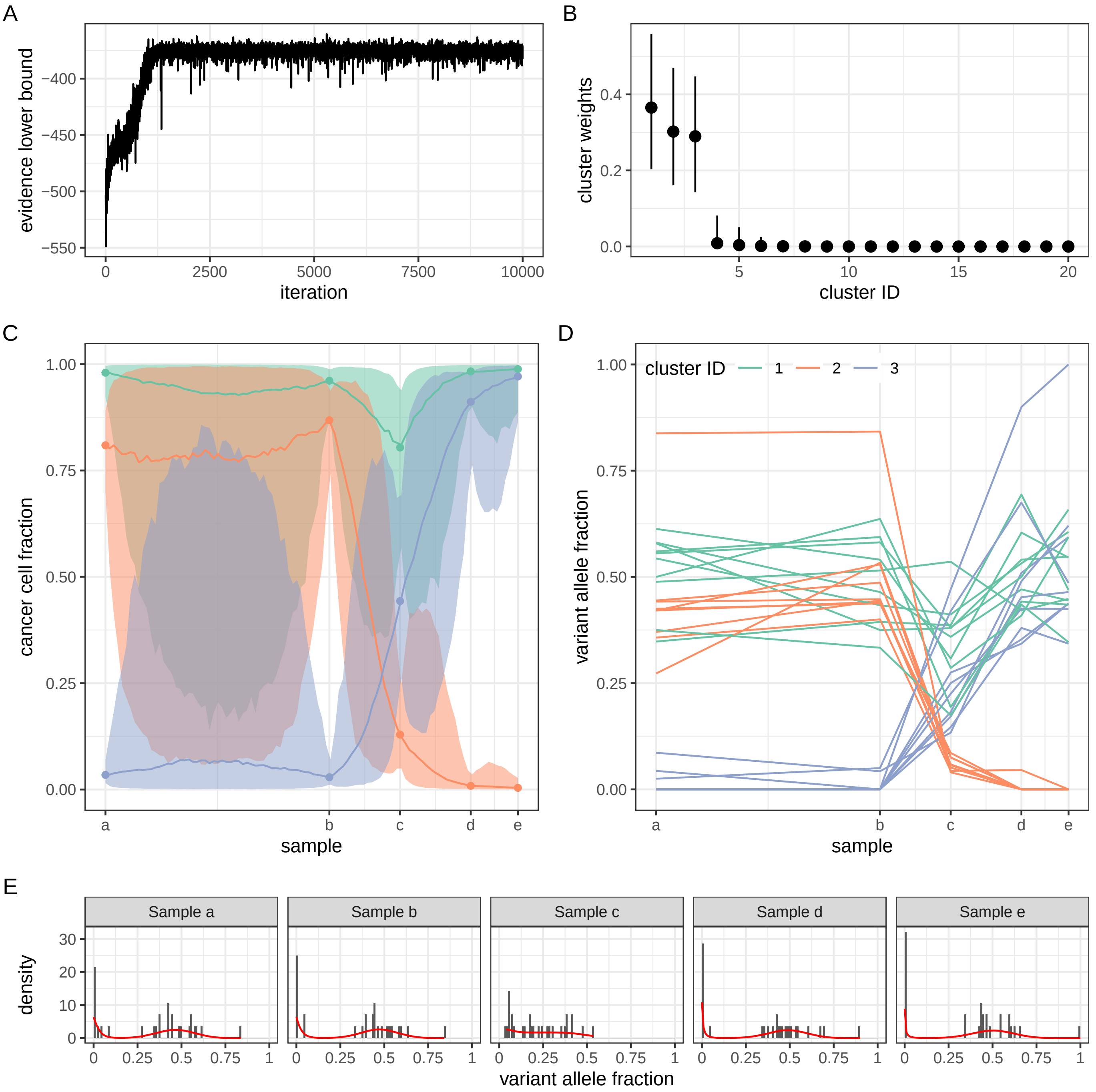

Figure 1: Application of model **GP0-Exp** on data from patient CLL003 [6]. A) Parameter estimation was achieved via maximisation of the evidence lowerbound. Convergence was attained in less than 3K iterations. B) The number of clusters in the data was automatically estimated using a Dirichlet Process prior. In this example, three major clusters were identified. C) The temporal profile of the three major clusters during disease treatment and progression. The median and 95% credible intervals are shown. Sample collection took place over the course of 35 months. D) Observed VAF values for each somatic mutation and their cluster assignment. E) The fitted model (red lines) against the data in each sample. Notice the wideer 95% credible intervals in comparison to other **GP0** models (Fig. 1 in the main text and Suppl. Figs. 2 and 3 below).

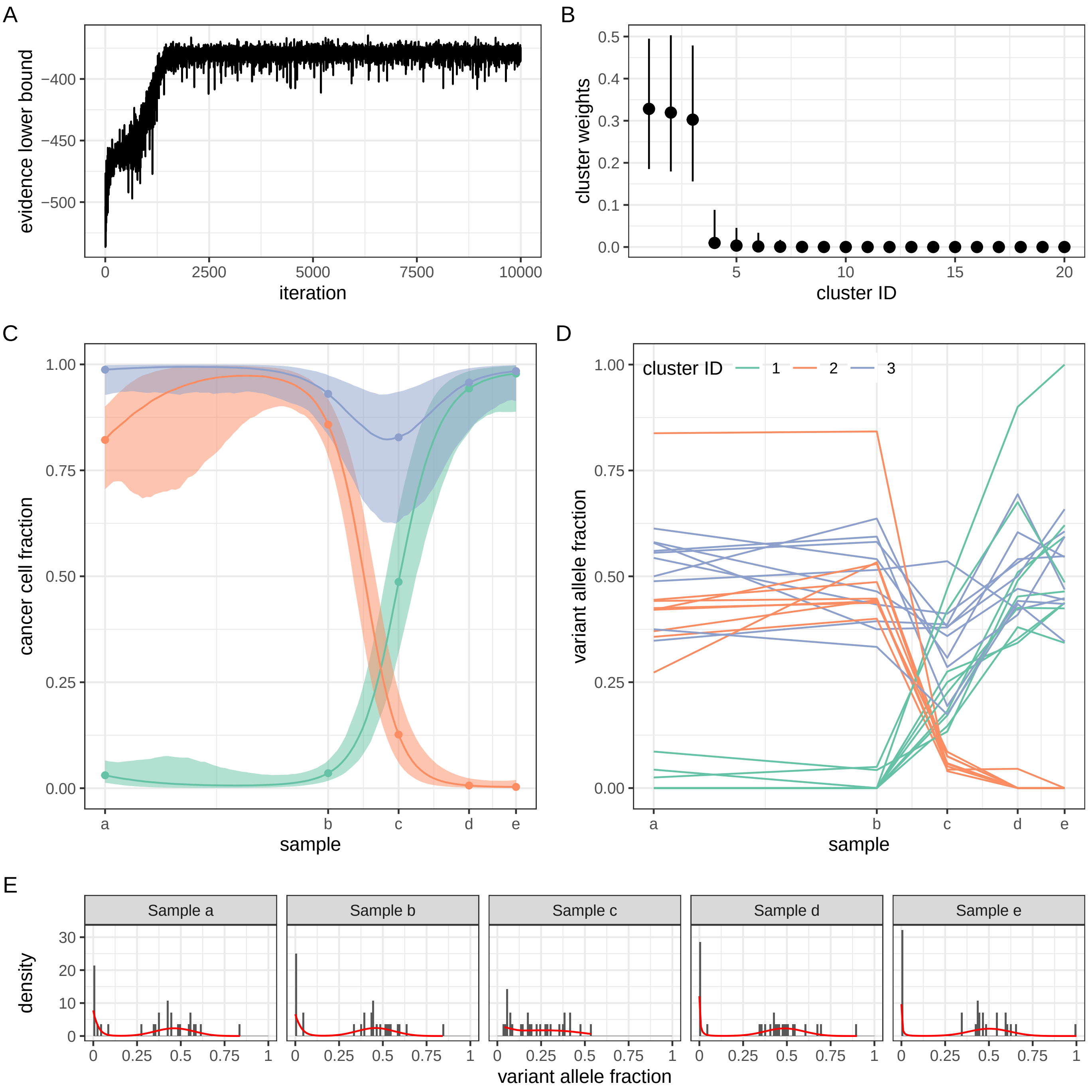

Figure 2: Application of model **GP0-Mat52** on data from patient CLL003 [6]. Description for panels A to E are as in the previous figure.

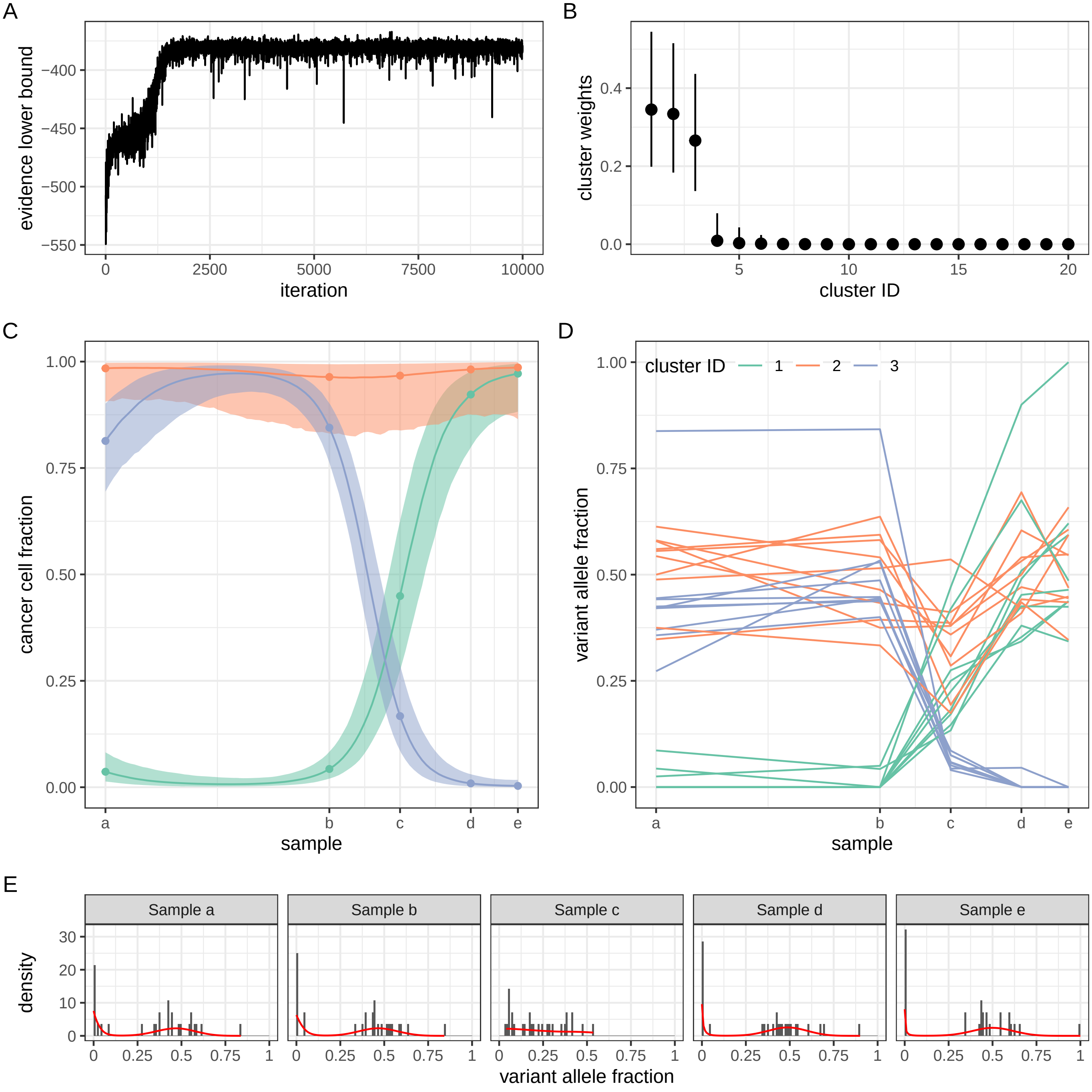

Figure 3: Application of model **GP0-ExpQ** on data from patient CLL003 [6]. Description for panels A to E are as in the previous figure.

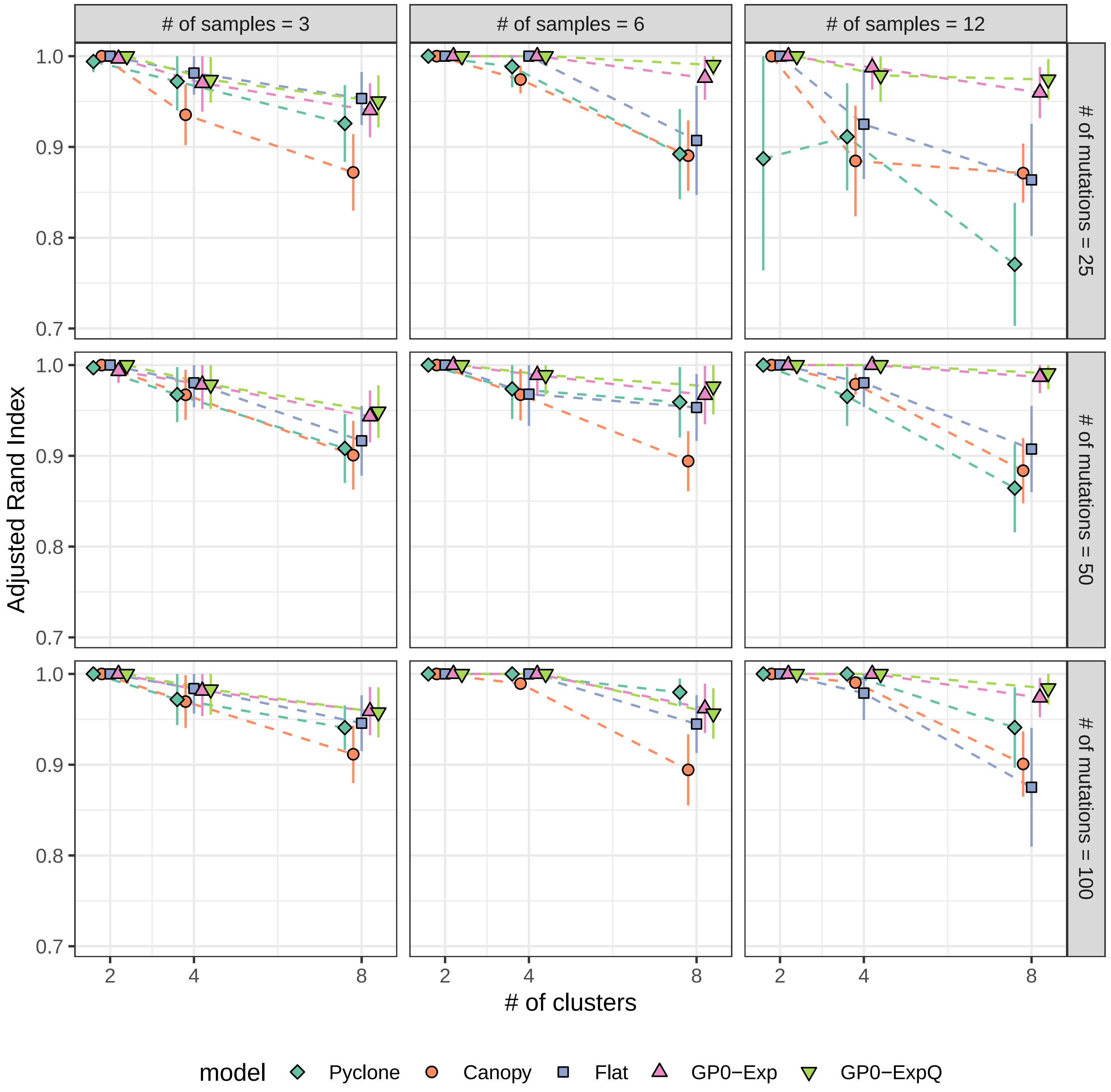

Figure 4: Benchmarks on synthetic data using **PyClone**, **Canopy** and **Flat** as baseline models. The two **GP0** models (**GP0-Exp** and **GP0-ExpQ**) are the only ones explicitly taking into account the temporal spacing of the longitudinally collected samples. See main text and Fig. 6 in the main text for more details. Also, see ***Supplementary Methods*** for details on how the synthetic data were generated.

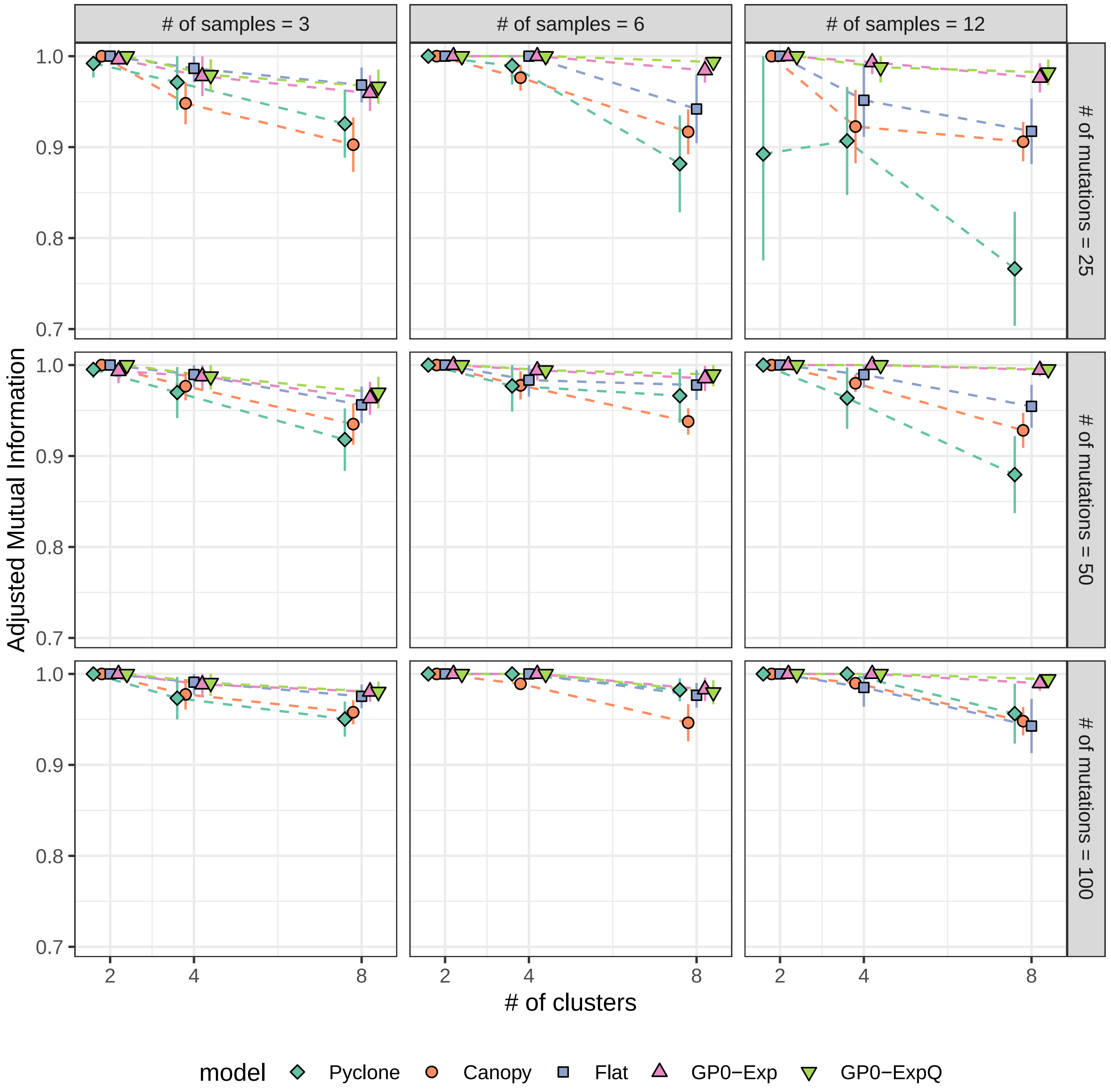

Figure 5: Benchmarks on synthetic data using **PyClone**, **Canopy** and **Flat** as baseline models and the *Adjusted Mutual Information* (AMI) as performance metric. See ***Supplementary Methods*** for calculation details.

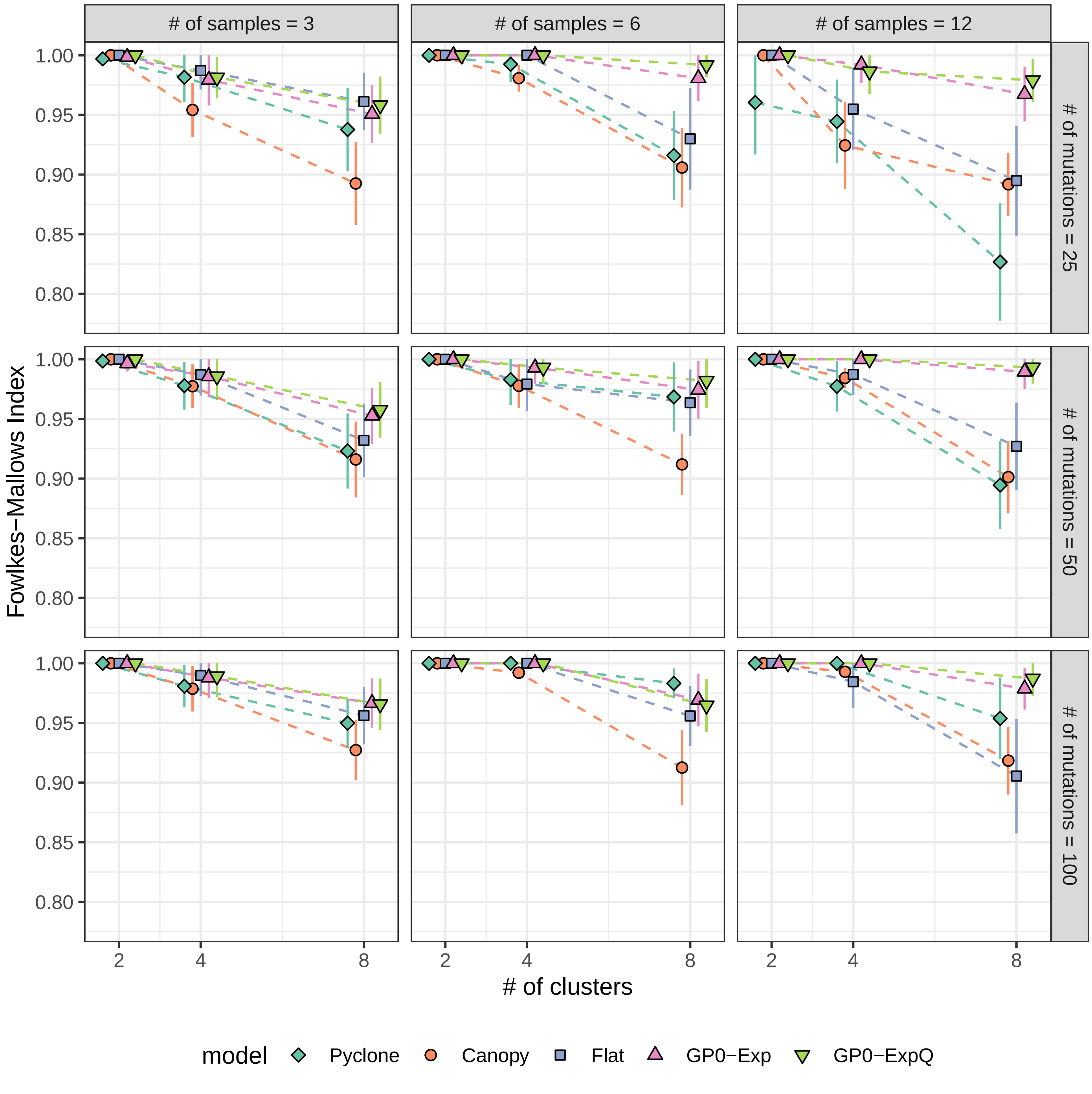

Figure 6: Benchmarks on synthetic data using **PyClone**, **Canopy** and **Flat** as baseline models and the *Fowlkes-Mallows Index* (AMI) as performance metric. See ***Supplementary Methods*** for calculation details.
